# Hospital Meetings

**Published:** 1906-06-30

**Authors:** 


### HOSPITAL MEETINGS.

### ST. GEORGE'S HOSPITAL

### THE QUESTION OF REMOVAL SHELVED ONCE MORE.

The Earl of Plymouth presided at a special Court of the Governors of St. George's Hospital on Tuesday, summoned "to consider offers for the purchase of the property and interest of St. George's Hospital at Hyde Park Corner." There was an attendance of some 50 or more Governors.

The Chairman said it would be recalled that at a special court held on July 14th, 1905, it was resolved, by 13 for to 7 against, "that the Secretary of the Hospital be, and is hereby, instructed forthwith to take such steps as the Treasurers may direct to make known the willingness of the Governors to consider offers for their property and interest at Hyde Park Corner, and to report to a special court to be held not later than June 1906, all resolutions of previous courts notwithstanding," and that meeting had accordingly been convened.

Mr. West, the Treasurer, then made the following statement: During the past year I have been trying to get definite offers for our interest in the site, and I have seen several would-be buyers. The trouble I have had to contend with has been that one and all have said, "What is the sum you want?" This question I have been unable to answer, and a good many of those who have been considering the matter have declined to make definite offers as the position now stands, saying, perhaps somewhat rightly, that if they did make an offer it would simply be used as an inducement to someone else to make a higher bid. My hands being tied under existing conditions, I have been unable to obtain an offer which I can recommend for acceptance to-day. I have two offers in writing made on behalf of responsible people who are known to me as such—the one in cash, the other in the form of an exchange of sites. The latter I cannot recommend, partly on the ground that the site is quite near one of the other large hospitals in London, and even then is not the best. It was only on Friday last that I heard definitely the minimum sum the Duke of Westminster's Estate Board would be willing to accept for their interest in the site. It is the large share asked for by the Duke's Board which makes this balance a comparatively small one, but the net result, after deducting the share asked for by the Duke's Estate Board, and repaying the £60,000 already paid for land in Knightsbridge, etc., would leave a net balance of about £270,000. This sum, with some negotiation, could be increased by some £10,000. I am going to ask this Special Court to-day to agree, first of all, that there is a sum at which, always subject to a suitable site being found, it would be desirable to move the hospital; and secondly, to fix a minimum price at which it would be desirable to move; and I am going to ask this because I am confident that if you will adopt this course much better offers can be obtained. I want to put clearly before you the alternative schemes, so that before recording your votes you may thoroughly understand the position of affairs. There are practically three alternatives to moving. The first is to continue your work with the hospital as it now stands, which will cost you £37,700; second, to buy up the leasehold interests of the houses in Knightsbridge and add to your present hospital it is estimated will cost £120,000; third, to rebuild the hospital on your present site will entail an expenditure of some £350,000. The minimum price I am going to suggest to you as one at which it would be desirable to move is a net amount of £400,000, arrived at as follows:—For a new hospital of 350 beds, at £600 per bed, £210,000; for a new site, £50,000; for law costs and contingencies, £50,000; together £310,000, leaving about £90,000 for an endowment fund, and our investments would remain intact. If, therefore, you can obtain £400,000 net, moving means, as compared with the first scheme, that you will not have to go to the public for £127,700; as compared with the second scheme a saving of £210,000; and as compared with the third scheme a saving of £440,000 (adding in each case the £90,000 endowment fund) Our available saleable stock is £204,000, and as our expenditure is greater than our income, it means we must ask the public to make up one or other of these sums. In conclusion I would only remind you that the whole staff has unanimously sent in a report saying that sooner or later the hospital will have to be rebuilt, and that they are averse to any building on the Knightsbridge site which will not form part of a new hospital: in other words, if you do not sell your site, sooner or later you must appeal to the public for £350,000. I am anxious there shall be no misunderstanding as to the view I take of this matter. I believe it would be to the interests of the patients of the hospital and the general public if a new hospital were built on a larger site and in the immediate neighbourhood of those whom we serve, and rather than have a scheme involving an outlay of £120,000 for additions to this hospital which can, to my mind, never be satisfactorily done, I would prefer to go to the public and ask them to give £120,000 to enable us to move.

I beg to move, first of all, that the Governors should place on record that in their opinion it is advisable that the hospital should remove to another site, provided a suitable one can be found at a certain figure. Then if that is carried I would move that the sum at which it would be desirable to move is a minimum of £400,000 for the hospital's interests.

Mr. Thorp seconded the resolution. It was obvious, he said, that they had not enough room where they were. If they stayed there they must rebuild, and that meant raising at least £350,000.

Mr. Harrison agreed with the previous speaker that they ought to move. When business people looked at their site it must occur to their minds that they were occupying one of the most expensive and extravagant positions in London, and that most of the money they wanted was in their cellars since the ground rent was worth £15,000 or £20,000 a year. If they could realise sufficient from the sale of their site to enable them to build a hospital on a suitable site elsewhere, it was their bounden duty to do so. He hoped before they voted against moving they would think carefully what was to be done to raise the money required if they stopped where they were. After all the hospital was for the poor, not for the squares near by, nor for accidents in the Park. If they could provide a better hospital in some convenient situation they ought to treat the proposition in a business-like way.

The Rev. R. J. Walker asked whether, in view of their trust deeds, it would be possible to move without a private Act of Parliament?

Lord Eustace Cecil, who observed that he had been a Governor nearly 50 years, said this question of removal had been discussed at the meeting two years ago, and a resolution had been carried against it. (Hear, hear.) He could not follow the Treasurer's figures, but he thought a fuller statement should be put before the Governors, the main body of whom were certainly not here. The figures laid before them were extremely formidable, and they could not be expected to decide at a moment's notice whether they would accept £310,000 or £320,000 for their site.

A point of order having been taken whether the resolution before the meeting was in order, the Chairman said he thought it was a good objection that the Governors had not had sufficient notice of the actual resolutions proposed. If objection was taken another meeting would have to be called if it was desired to consider them. The complexity of the subject was great, and the variety of resolutions passed from time to time were not altogether consistent. He ruled the resolution out of order.

Mr. C. G. Heathcote said that two years ago, by a very large majority, they decided not to move, and he thought that at least they were entitled to have before them a definite offer of a definite sum.

Mr. West said he had submitted a definite offer which had been made to him in writing, the net result of which would be to the hospital £270,000. He did not think that sum was adequate, and it was with a view to getting a better one that he submitted his resolution.

The Duke of Grafton asked whether the resolution of two years ago was rescinded?

The Chairman replied that it was so in effect by the resolution of last July which had been read, expressing the willingness of the Governors to consider offers.

Mr. Timothy Holmes said he believed the keeping open of the question of removal was doing an immense amount of injury to the hospital and to the medical school. (Hear, hear.) If it were in order he should like to move that in no circumstances at present known to them should the hospital be moved from its present site. (Hear, hear.)

In reply to questions the Chairman said the resolution passed by the meeting at the Westminster Palace Hotel on June 21st, 1904, declared that it was not desirable under existing circumstances to move the hospital.

Mr. Warrington Haward asked if it would be in order to move that the meeting had been called to consider offers for the purchase of the property; that these offers were not adequate, and that the matter be no further considered. It was certainly detrimental that the question should be left open, both for the hospital and the school. He had originally been in favour of moving, but it seemed now hardly possible that a site could be obtained within reasonable distance. He therefore hoped they would come to a decision that the matter be dropped.

Sir Theodore Hope and other Governors having spoken,

Dr. Latham said he understood the position at the last meeting to be that if by the end of June no acceptable offer had been received, then the whole matter would be concluded. (Hear, hear.) What they had to consider was whether an offer yielding £270,000 would justify them in moving from what was, from the medical point of view, the only proper site for a hospital in London.

The Duke of Grafton suggested that perhaps the best way to give effect to the evident sense of the meeting would be to reaffirm the resolution come to two years ago, and this was seconded by Lord Eustace Cecil.

Mr. Haward pointed out that this would still leave some uncertainty, since that resolution said "under existing circumstances."

Mr. Harrison thought it would be unwise to bind the Governors to the proposition that they were never to move.

The Chairman suggested that the Duke of Grafton's and Mr. Haward's resolutions should be combined to read that "the meeting having had no adequate offer put before it, the resolution of June 21st, 1904, be reaffirmed," and this suggestion was accepted.

Mr. Thorp desired to add as a rider "but that if a further offer be obtained by the Treasurer which will yield £400,000 after payment of the Duke of Westminster and for the houses in Knightsbridge, such offer may be brought before the Court of Governors."

Colonel Britton seconded this rider, which, however, was lost on being put to the meeting, only six hands being held up for it, and Mr. Haward's resolution was then adopted *nem. con.*

Mr. Timothy Holmes hoped the Governors might go home with the comfortable assurance that the question of moving was now finally buried; but Mr. West pointed out that no resolution could bind the hands of the Governors for ever and ever, and that the question of what they were to do if they stayed where they were would have to be considered eventually.

The meeting then separated.

